# Anxiety, Depression, and Suicidality Among Testicular Cancer Survivors

**DOI:** 10.1002/cam4.71602

**Published:** 2026-02-10

**Authors:** Margaret Meagher, Paul Riviere, Tyler Nelson, Kylie Morgan, Dhruv Puri, Kshitij Pandit, Nuphat Yodkhunnatham, Kit Yuen, Jacob Taylor, Daniel Herchenhorn, Tyler Stewart, Juan Javier‐Desloges, Amirali Salmasi, Rana McKay, Sean Kern, Fred Millard, Brent Rose, Aditya Bagrodia

**Affiliations:** ^1^ Department of Urology UC San Diego School of Medicine La Jolla California USA; ^2^ Department of Radiation Medicine and Applied Sciences UC San Diego School of Medicine La Jolla California USA; ^3^ VA Hospital San Diego San Diego California USA; ^4^ Department of Urology UTSW School of Medicine Dallas Texas USA; ^5^ Department of Urology Uniformed Services University and Walter Reed Army Medical Center Bethesda Maryland USA

**Keywords:** anxiety, depression, suicidality, testicular cancer

## Abstract

**Introduction:**

We evaluated the incidence of anxiety, depression, and suicidality amongst TC survivors and the impact of chemotherapy on these outcomes.

**Methods:**

We conducted a retrospective cohort study of men diagnosed with TC in the United States Veterans Affairs Health System from 1990 to 2016. De novo anxiety or depression was a composite endpoint comprised of diagnosis codes for anxiety, depression, or medications used to treat these diagnoses. Incident suicidality was defined as a diagnosis code for suicidal ideation. 2022 TC patients were compared in a 1:3 ratio to 6375 controls. Cox proportional hazards models were employed for statistical analysis.

**Results:**

Mean age at diagnosis was 42.6 years. 5‐year cumulative incidence of anxiety or depression was 53.4% in TC patients and 35% for controls (*p* < 0.001). TC patients were more likely to develop anxiety or depression (HR 1.66, 95% CI 1.56–1.78, *p* < 0.001) and suicidality (HR 22.99, 95% CI 17.52–30.17, *p* < 0.001). In the TC cohort, factors associated with a higher risk of anxiety or depression were divorce (HR 1.15, 95% CI 1.00–1.32, *p* = 0.044), unemployment (HR 1.68, 95% CI 1.47–1.9, *p* < 0.001), and receipt of chemotherapy (HR 1.20, 95% CI 1.06–1.35, *p* < 0.001).

**Conclusions:**

Psychological morbidity due to depression, anxiety, and suicidality is high among TC survivors. In our analysis chemotherapy increases the rates of psychosocial morbidity. Clinicians should be proactive in screening and intervening for these diagnoses in TC survivors to provide early intervention and improve health comes.

## Graphical abstract

Anxiety, depression, and suicidality rates are high amongst testicular cancer survivors. Chemotherapy appears to increase the risk of new development of adverse mental health outcomes for these patients.

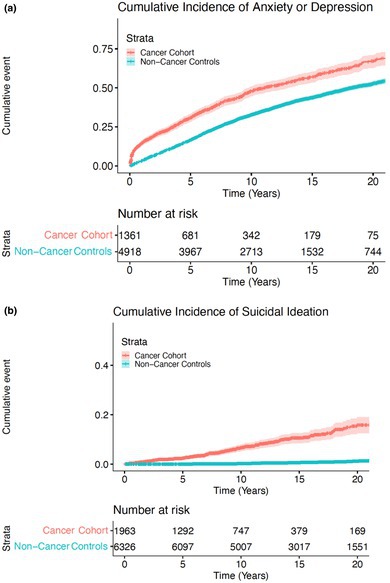

## 1 Introduction

Testicular cancer (TC) represents the most common solid malignancy diagnosis among men ages 15–44 years old, and is the most common malignancy in active duty service members who ultimately transition care to the Veteran's Association (VA) [1, 2]. In the United States, there is an incidence of about 10,000 cases yearly with average age at diagnosis of 33 [1]. Fortunately, advancements in surgical and systemic therapy options have resulted in survivorship rates above 95% [2].

TC is usually cured surgically in the case of localized disease or by chemotherapy with or without surgery in the case of metastatic disease. Platinum‐based chemotherapy regimens are standard, with high‐rates of complete responses, even in the metastatic setting. However platinum agents are associated with both short and long‐term toxicities [3, 4], including cardiopulmonary disease, ototoxicity, neurotoxicity, and decreased fertility [5, 6].

However, research regarding psychosocial and mental health sequelae of chemotherapy is more nebulous. Several studies done on TC survivors have found that the psychosocial aspects of the disease, such as anxiety or work issues, are more important determinants of distress, morbidity and QoL than the specific type of treatment undergone or even the time since its completion [7]. It is thus possible that subjective evaluations of mental health are more important determinants of functioning and overall distress than actual medical history [7]. Specifically, to our knowledge no study has examined the impact of chemotherapy upon development of poor mental health outcomes, including anxiety, depression, and suicidality, in the TC population.

Given the young average age at diagnosis and high probability of cure, survivorship issues represent an important source of treatment burden [5, 8]. TC is an excellent model to evaluate not only long‐term survival but also quality of life and psychological disorders in survivors. These features, along with all Adolescent Young Adult cancers, lead to unique considerations that differ from malignancies arising later in life that are usually associated with competing comorbidities and ambient exposures. Such problems, including individual health, sexual relationships, and work problems, affect several important aspects of survival and significantly influence the quality of life (QoL) of long‐term survivors.

We sought to evaluate the impact of chemotherapy on anxiety, depression, and suicidality amongst TC survivors in the veteran population; evaluate the impact of treatment modalities among TC survivors; and compare rates of anxiety, depression, and suicidality between TC and non‐cancer controls. We hypothesize that rates of anxiety, depression, and suicidality will be higher in the TC patient population. We hope that these results may improve treatment for TC survivors by encouraging providers to screen for mental health outcomes in this population and offer early intervention as indicated.

## 2 Methods

### 2.1 Data Source

This was a retrospective study utilizing data from the Veterans Affairs (VA) national electronic health record, the Corporate Data Warehouse, and the VA Central Cancer Registry accessed through the VA Informatics and Computing Infrastructure (VINCI). Informed consent was waived due to the retrospective nature of this study and the minimal risk posed to patients. The VA Central Cancer Registry conforms to standards set by the North American Association of Central Cancer Registries for detecting and reporting incident cancer cases and treatments. Approval for this study was granted by the San Diego VA Institutional Review Board.

### 2.2 Study Population

We initially identified 2824 patients with a diagnosis of TC from ICD 10 codes. Patients were excluded based on non‐germ cell tumor histology (*n* = 247), incomplete staging (*n* = 527), and incomplete follow‐up data (*n* = 28), to yield a final cohort of 2022 patients. These were age‐ and race‐matched to a control group of 6375 patients without TC. Because of the nature of requesting data in VINCI, the 2824 patients identified with testicular cancer were exact matched at a ratio of 1:5 on birth year and race. Since exact matching was used, not every cancer patient had 5 non‐cancer patients matched to them; this led to a cohort of 7595 matched patients. The diagnosis information of these 7595 patients were then added to the IRB's VINCI space. Further cleaning based on exclusion criteria occurred after patients' data was moved into VINCI space.

Demographics, including race (white, other, Native American, African American, Asian Pacific Islander), ethnicity (Hispanic, non‐Hispanic), marital status (single, married, divorced/separated/widowed), tobacco use history (current, former, never, unknown), and employment status (employed, unemployed, retired) were obtained from the national health record. Cancer information including, year of diagnosis, patient's age at diagnosis, clinical T staging (T1, T2, T3, T4), clinical N staging (N0, N1, N2, N3), clinical M staging (M0, M1), and treatment were obtained from the cancer registry.

### 2.3 Outcomes

The primary outcome was development of de novo anxiety or depression which was a composite endpoint comprised of International Statistical Diagnosis Classification of Diseases and Related Health Problems (ICD) codes for anxiety (ICD F41.9, F41.1, F43.3), depression (ICD F32.0, F32.1, F32.2, F32.2, F32.3, F32.9, F33, F33.1, F33.2, F33.3, F33.9) or administration of medications used to treat these diagnoses for at least 6 months duration [8, 9]. These medications are included as Table S1. For statistical analyses, anxiety and depression diagnoses and medication use were collapsed into a single composite binary outcome. Patients were classified as having the outcome at the time of first qualifying diagnosis or medication exposure, and only the first event was considered in time‐to‐event analyses. The secondary outcome was development of de novo suicidality which was defined as diagnosis code for suicidal ideation (R45.851). Time to event was defined as time from diagnosis to event or censored at the time of last follow‐up. Rates of outcomes were reported through cumulative incidences.

### 2.4 Statistical Analysis

To compare differences between TC patients and non‐cancer controls, a chi‐squared test was performed (Table 1). Continuous variables were evaluated with Wilcoxon rank‐sum tests (Table 1). We employed Cox proportional hazards models with clustering for matched analysis with non‐cancer controls (Tables 2a and 2b). Demographic variables, including age, race, marital status, employment status, stage at diagnosis, and year of diagnosis, were adjusted for the in the Cox proportional hazards models. Multicollinearity was evaluated using Pearson correlations among continuous variables and variance inflation factors (VIFs) derived from the Cox models. Adjusted GVIF values for categorical predictors were all near 1.0–1.06, showing no evidence of problematic collinearity.

**TABLE 1:** demographics and clinical characteristics of cancer cohort.

Variable	Cancer cohort (*n* = 2022, %)
Mean age at diagnosis (years, SD)	42.46 (13.40)
Clinical stage	
I	1485 (73.4%)
II	275 (13.6%)
III	261 (12.9%)
Clinical T stage	
1	18 (0.9%)
2	1258 (62.2%)
3	397 (19.6%)
4	89 (4.4%)
Null	14 (0.7%)
Clinical N stage	
0	1406 (69.5%)
1	145 (7.2%)
2	143 (7.1%)
3	101 (5.0%)
Null	227 (11.2%)
Clinical M stage	
0	1632 (80.7%)
1	197 (9.7%)
Null	193 (9.5%)
Chemotherapy	685 (33.9%)
Radiation	507 (25.1%)
Surgery	1957 (96.8%)
Treatment regimen	
Chemotherapy alone	36 (1.8%)
Radiation alone	11 (0.5%)
Surgery alone	840 (41.5%)
Radiation + chemotherapy	3 (0.1%)
Surgery + chemotherapy	624 (30.9%)
Surgery + radiation	471 (23.2%)
Surgery + radiation + chemotherapy	22 (1.1%)
Unknown	15 (0.7%)
Race	
White	1814 (89.7%)
Black	121 (6.0%)
Native	14 (0.7%)
Asian/pacific islander	25 (1.2%)
Other	48 (2.4%)
Hispanic	125 (6.2%)
Employment status	
Employed	745 (36.8%)
Unemployed	926 (45.8%)
Retired	239 (11.8%)
Unknown	112 (5.5%)
Smoking status	
Current	808 (40.0%)
Never	587 (29.0%)
Prior	337 (16.7%)
Unknown	290 (14.3%)
Year diagnosed	
1990–1999	403 (19.9%)
2000–2009	699 (34.6%)
2010–2021	920 (45.5%)

**TABLE 2a:** Cox proportional hazards model of matched cohort for de novo anxiety or depression.

Variable	HR	95% CI	*p*
Cancer (vs. non‐cancer)	1.66	1.56–1.78	< 0.001
Age	1.01	1.00–1‐1.01	< 0.001
Race (referent Asian/Pacific Islander)			
Black	0.88	0.53–1.45	0.61
Native	0.70	0.28–1.76	0.45
White	0.90	0.55–1.47	0.67
Other/unknown	0.88	0.53–1.48	0.64
Year diagnosed (vs. 1990–1999)			
2000–2009	1.79	1.65–1.94	< 0.001
2010–2021	2.65	2.44–2.87	< 0.001

**TABLE 2b:** Cox proportional hazards model of matched cohort for de novo suicidality.

Variable	HR	95% CI	*p*
Cancer (vs. non‐cancer)	22.99	17.52–30.17	< 0.001
Age	1.00	0.99–1.01	0.638
Race (referent Asian/Pacific Islander)			
Black	1.07	0.26–4.37	0.92
Native	1.64	0.22–11.95	0.63
White	1.02	0.28–3.78	0.97
Other/unknown	0.65	0.13–3.25	0.60
Year diagnosed (vs. 1990–1999)			
2000–2009	6.43	3.7–11.17	< 0.001
2010–2021	22.99	11.76–37.81	< 0.001

The proportional hazards assumption was assessed using scaled Schoenfeld residuals obtained from the clustered Cox models (cox.zph). Both global and covariate‐specific tests were examined, and residual plots were visually inspected for systematic temporal trends. The global tests showed no statistically significant departures from proportionality in the final adjusted models.

**FIGURE 1:**
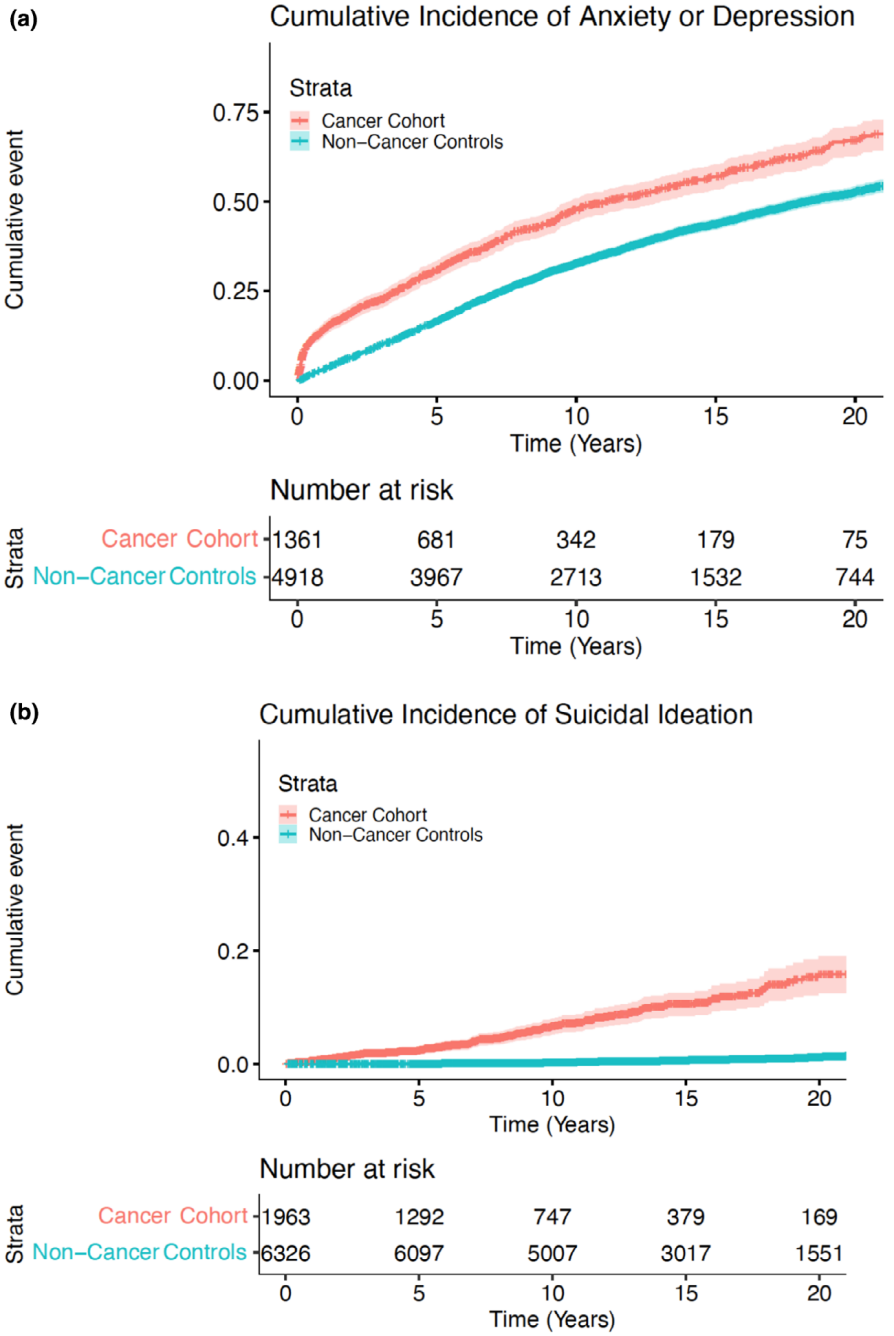
(a) Cumulative incidence of de novo depression or anxiety by cancer diagnosis. (b) Cumulative incidence of de novo suicidality by cancer diagnosis.

**FIGURE 2:**
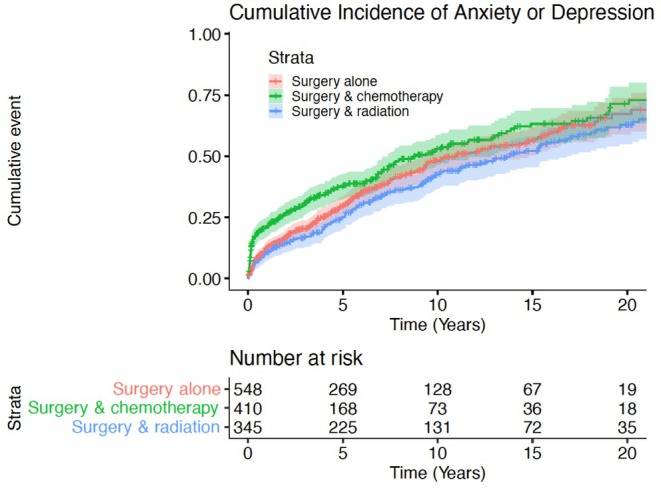
Cumulative incidence of de novo depression or anxiety by treatment.

**FIGURE 3:**
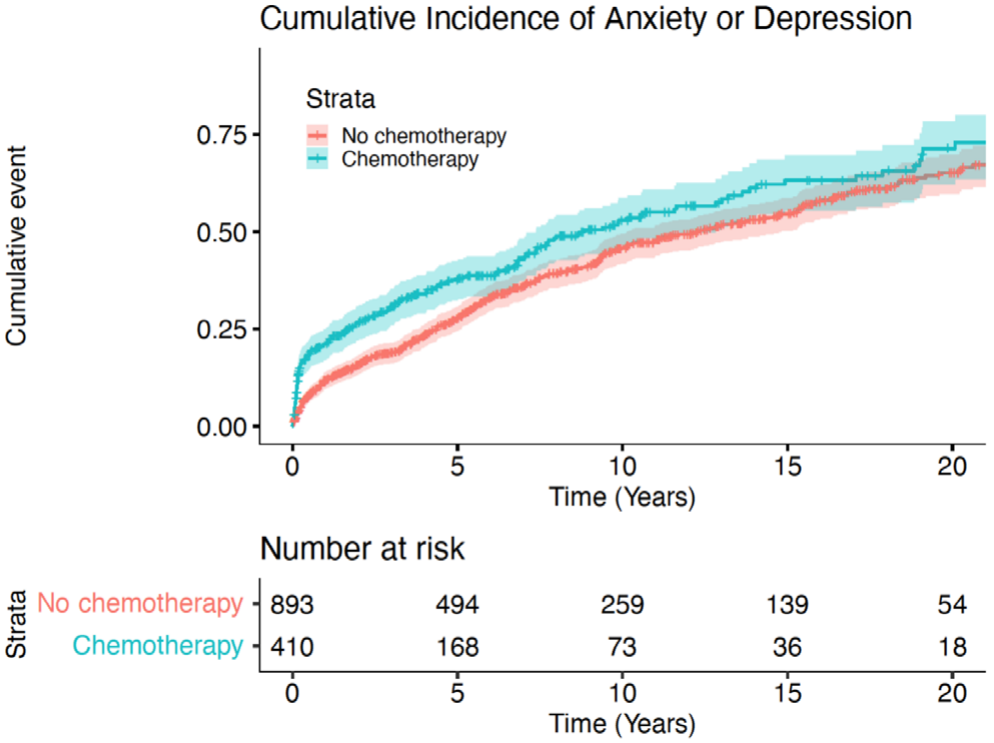
Cumulative incidence of de novo depression or anxiety by receipt of chemotherapy.

We then conducted a cox proportional hazards model in the TC cohort (Table 2c). To evaluate the impact of treatment regimen on outcomes, we then conducted a cox proportional hazards analysis of all TC patients based on receipt of chemotherapy (Table 2d). Estimated hazard ratios (HRs) and associated 95% confidence intervals (CI) were reported. Cumulative incidences were calculated to establish rates of primary and secondary outcomes (Figures 1, 2, 3). Patients with previous history of depression, anxiety, or suicidality were not included in de‐novo analysis. All statistical tests were two‐sided, with significance defined as *p* < 0.05, and analysis was performed using R Studio version 3.5.1 (The R Foundation, Vienna, Austria).

**TABLE 2c:** Cox proportional hazards model of cancer cohort for de novo anxiety or depression.

Variable	HR	95% CI	*p*
Age	1.01	1–1.01	0.623
Treatment (reference surgery only)			
Surgery + chemotherapy Surgery + radiation	1.18 0.97	1.01–1.37 0.83–1.12	0.035 0.648
Race (referent Asian/Pacific Islander)			
Black	0.76	0.44–1.31	0.32
Native	0.66	0.26–1.69	0.39
White	0.80	0.49–1.32	0.38
Other/unknown	0.81	0.44–1.51	0.51
Stage at diagnosis (referent I)			
Stage II	0.98	0.82–1.17	0.78
Stage III	0.88	0.71–1.09	0.24
Marital status (referent married)			
Divorced/separated/widowed Unmarried	1.15 0.81	1–1.32 0.71–0.94	0.04 0.01
Employment status (referent employed)			
Unemployed	1.68	1.47–1.90	< 0.001
Retired	1.50	1.22–1.85	< 0.001
Unknown	1.26	0.97–1.63	0.09
Year diagnosed (vs. 1990–1999)			
2000–2009	2.16	1.81–2.57	< 0.001
2010–2021	3.01	2.52–3.60	< 0.001

**TABLE 2d:** Cox proportional hazards model cancer cohort by receipt of chemotherapy for de novo depression or anxiety.

Variable	HR	95% CI	p‐value
Chemotherapy	1.19	1.03–1.37	0.015
Age	1.01	1–1.01	0.629
Race (referent Asian/Pacific Islander)			
Black	0.75	0.44–1.30	0.309
Native	0.66	0.26–1.70	0.393
White	0.80	0.49–1.31	0.374
Other/unknown	0.81	0.44–1.50	0.506
Stage at diagnosis (referent I)			
Stage II	0.97	0.81–1.16	0.741
Stage III	0.88	0.71–1.08	0.231
Marital status (referent married)			
Divorced/separated/widowed Unmarried	1.15 0.81	1–1.31 0.71–0.94	0.045 0.005
Employment status (referent employed)			
Unemployed	1.68	1.48–1.91	< 0.001
Retired	1.50	1.22–1.85	< 0.001
Unknown	1.26	0.97–1.63	0.085
Year diagnosed (vs. 1990–1999)			
2000–2009	2.16	1.82–2.58	< 0.001
2010–2021	3.04	2.55–3.62	< 0.001

## 3 Results

### 3.1 Patient Characteristics

2022 patients with TC and 6375 non‐cancer control patients were analyzed. Mean age at TC diagnosis was 43 years for the cancer cohort, and ages ranged from 18 to 88 years old. 32.4% of TC patients and 22.9% of non‐cancer patients had a prior history of anxiety or depression (*p* < 0.001).

Table 1 displays demographic and oncologic descriptive variables for the TC patients. 73.4% of patients were clinical stage I, 13.6% had clinical stage II, and 12.9% had clinical stage III at diagnosis. 840 (41.5%) of patients underwent surgery (orchiectomy) alone, 471 (23.3%) underwent surgery and radiation, 624 (30.9%) underwent surgery and chemotherapy, and 87 (4.2%) of patients underwent other treatment (surgery + chemotherapy + radiotherapy). 1339 patients (66.3%) had primary seminoma histology.

Of the 1277 patients who were identified as having anxiety or depression, 914 patients had at least one ICD code of anxiety or depression, 1122 patients had at least 6 months of drug prescription, and 749 patients met both criteria.

### 3.2 Matched Case–Control Analyses

On clustered univariable Cox regression analysis, TC diagnosis (vs. non‐cancer, HR 1.73, *p* < 0.001) was associated with increased cumulative incidence of de novo anxiety or depression. In multivariable regression correcting for age, race, and year of diagnosis, TC survivors had a HR of 1.66 (1.56–1.78, *p* < 0.001) for development of de novo anxiety or depression. Additionally, increasing age (HR 1.01, 95% CI 1.00–1.01, p < 0.001) and more recent year of diagnosis (years 2000–2009 HR 1.79, 95% CI 1.65–1.94, *p* < 0.001; years 2010–2021 HR 2.65; 95% CI 2.44–2.87, p < 0.001) were also associated with increased cumulative incidence of de novo anxiety or depression (Table 2a). At 5 years, cumulative incidence of anxiety or depression was 53.3% for TC patients and 35.2% for controls (*p* < 0.001). 5‐year incidence of de novo depression or anxiety was 30.1% for TC patients and 16.7% for controls (*p* < 0.001) (Figure 1a).

TC diagnosis (vs. non‐cancer, HR 22.99, 95% CI 17.52–30.17, *p* < 0.001) and more recent date of diagnosis (2000–2009 vs. 1990–1999 HR 6.43, 95% CI 3.70–11.17, *p* < 0.001; 2010–2021 vs. 1990–1999 HR 21.09, 11.76–37.81, p < 0.001) were associated with increased incidence of suicidality (Table 2b). 5‐year cumulative incidence of suicidality was 5% for TC patients and 0.1% for controls (*p* < 0.001). De novo incidence was 2.4% for TC patients and 0.1% for controls (Figure 1b).

### 3.3 Cancer‐Specific Factors

Cox regression analysis demonstrated receipt of combination chemotherapy and surgery (compared to surgery alone, HR 1.18, 95% CI 1.01–1.37, *p* = 0.035), more recent date of diagnosis (2000–2009 vs. 1990–1999 HR 2.16, 95% CI 1.81–2.57, *p* < 0.001; 2010–2021 vs. 1990–1999 HR 3.01, 95% CI 2.52–3.60, *p* < 0.001), divorced status (referent married HR 1.15, 95% CI 1.00–1.32, *p* = 0.044), and unemployment at time of diagnosis (compared to employed HR 1.68, 95% 1.47–1.90, *p* < 0.001) or retired status (referent employed HR 1.50, 95% CI 1.22–1.85, p < 0.001) to be associated with increased risk of development of depression or anxiety (Table 2c). 5‐year cumulative incidence of anxiety or depression was 50% for surgery alone, 56% for surgery and chemotherapy, 41% for surgery and radiation, and 45% for surgery, radiation, and chemotherapy (Figure 2). Across all TC patients, receipt of chemotherapy (referent no chemotherapy) was independently associated with increased risk of development of depression or anxiety (HR 1.19, 95% CI 1.03–1.37, *p* = 0.015) (Table 2d). 5‐year cumulative incidence of anxiety or depression was 57% for patients who received chemotherapy and 47% for those that did not (*p* < 0.001) (Figure 3). There was no impact of treatment modality or receipt of chemotherapy on development of suicidality.

## 4 Discussion

In a large, diverse, multicenter cohort, we found that rates of anxiety, depression, and suicidality were higher in TC patients compared to age‐matched peers. Notably, receipt of chemotherapy was independently associated with a greater risk of anxiety and depression compared to other treatment modalities independent of stage. Despite its oncologic efficiency, chemotherapy is associated with increased rates of psychosocial morbidity for TC patients. Thus, chemotherapy may confer a heightened survivorship burden that requires identification and intervention by clinicians and healthcare professionals. These results are especially timely given that rates of depression, anxiety, and suicidality appear to be increasingly prevalent in contemporary cohorts [10].

As life expectancy for a variety of malignancies has improved with refinement of surgical techniques, staging, and the development of novel therapeutics, new challenges regarding caring for patients post oncologic treatment have emerged [11, 12]. The concept of cancer survivorship, encapsulating the long‐term physical, financial, and psychological effects of cancer treatment, has garnered recent attention. Earle et al., in a study of 1111 cancer survivors compared to 4444 controls, identified that cancer survivors had significantly more general outpatient medical visits (27.4 vs. 21.9, *p* < 0.001), and mental health visits in particular (2.5 vs. 1.7, *p* < 0.001). This increased rate of mental health care utilization has important implications for both physicians and policymakers who must address access to care for an ever‐increasing number of cancer survivors.

Previous literature has attempted to capture the mental health sequala experienced by TC survivors. Smith et al. identified a greater prevalence of clinically significant anxiety, but not depression, in TC survivors in a meta‐analysis of 36 studies [13]. In particular, approximately 30% of survivors experienced fear of cancer return. In an analysis of 2619 surgically treated patients with TC compared to 13,095 control patients, Raphael et al. determined that patients with TC were significantly more likely to require peri‐treatment (aRR 2.45, 95% CI 2.06–2.92) and post‐treatment (aRR 1.3, 95% CI 1.12–1.52) mental health services, which persisted over the follow‐up duration [14].

Ours is the first study to identify receipt of chemotherapy as an independent risk factor for the development of anxiety and depression. Our analysis is the first to allow for population‐level data within an equal access health system while also providing access to granular level data regarding medications and subsequent diagnoses. While controlling for cancer stage, we detected that patients who underwent chemotherapy and surgery had rates of anxiety and depression in excess of patients who underwent surgery alone or combination treatment with surgery and radiation. A potential mechanism for this excess may be related to the treatment burden of chemotherapy which requires multiple treatments that is often associated with both physical and financial toxicity [15]. While chemotherapy‐induced cognitive effects and depression have previously been associated with taxane‐based therapy for breast cancer, there has been no such investigation of the mechanistic interplay of platinum‐based therapy and maladaptive neurobiology [16]. As TC patients are often young and otherwise healthy men, negative mental health outcomes may also be related to treatment‐induced infertility and hypogonadism. As we do not have information regarding treatment of infertility or hypogonadism, we are unable to distinguish the underlying cause of mental health deterioration. Our study calls for early incorporation of psychosocial therapy into comprehensive treatment for patients with TC [17, 18, 19].

Our study is limited by the inherent biases introduced in a retrospective study design, which is exacerbated by variation in practice patterns and data collection captured by a multi‐center database. We did include administration of medications as a proxy for primary and secondary outcomes, which may have introduced bias as these medications can also be utilized to treat a variety of other non‐psychiatric conditions. Additionally, we do not have information on other factors that may influence mental health outcomes including education, income, lack of ability to pursue employment, and presence of children, which may influence our results. We also lack information regarding receipt of treatment at high‐volume centers, which has been previously demonstrated to positively impact survival outcomes [20]. Our population age is older due to the requirements of entrance to the VA system (i.e., no longer active‐duty servicemen). Additionally, the baseline rates of anxiety, depression, and suicidality are high in the VA population which may limit ability to apply results to the civilian population. However, our data also underscores the pressing mental health needs that may be unique to the military population. The population receiving chemotherapy typically has more advanced disease and may often require additional post‐chemotherapy surgery to remove residual tumor, which may independently affect the endpoints measured here. Despite these potential limitations, we present outcomes from a large cohort of patients with TC that suggest cancer treatment and specifically chemotherapy increases rates of psychosocial morbidity [21, 22].

## 5 Conclusion

Psychosocial morbidity is high among TC survivors. Despite being effective and necessary for maintaining excellent oncologic outcomes, chemotherapy is associated with increased rates of psychosocial morbidity. Clinicians should be proactive in identifying TC survivors at risk for developing adverse mental health outcomes and consider implementation of multidisciplinary care with behavioral health interventions.

## Author Contributions

**Margaret Meagher:** writing – original draft (equal), writing – review and editing (equal). **Paul Riviere:** conceptualization (equal), data curation (equal), formal analysis (equal), methodology (equal). **Tyler Nelson:** conceptualization (equal), data curation (equal), formal analysis (equal). **Kylie Morgan:** data curation (equal), formal analysis (equal). **Dhruv Puri:** writing – review and editing (equal). **Kshitij Pandit:** writing – review and editing (equal). **Nuphat Yodkhunnatham:** writing – review and editing (equal). **Kit Yuen:** writing – review and editing (equal). **Jacob Taylor:** writing – review and editing (equal). **Daniel Herchenhorn:** writing – review and editing (equal). **Tyler Stewart:** writing – review and editing (equal). **Juan Javier‐Desloges:** writing – review and editing (equal). **Amirali Salmasi:** writing – review and editing (equal). **Rana McKay:** writing – review and editing (equal). **Sean Kern:** writing – review and editing (equal). **Fred Millard:** writing – review and editing (equal). **Brent Rose:** conceptualization (equal), investigation (equal), project administration (equal), supervision (equal), writing – review and editing (equal). **Aditya Bagrodia:** conceptualization (equal), project administration (equal), supervision (equal), writing – review and editing (equal).

## Funding

The authors have nothing to report.

## Ethics Statement

Informed consent was waived due to the retrospective nature of this study and the minimal risk posed to patients. The VA Central Cancer Registry conforms to standards set by the North American Association of Central Cancer Registries for detecting and reporting incident cancer cases and treatments. Approval for this study was granted by the San Diego VA Institutional Review Board. This study was performed in accordance with the Declaration of Helsinki.

## Consent

The authors have nothing to report.

## Conflicts of Interest

The authors declare no conflicts of interest.

## Supporting information


**Table S1:** cam471602‐sup‐0001‐TableS1.docx.

## Data Availability

Data is available upon request from the corresponding author.
