# Supplementary material for: Anxiety, Depression, and Suicidality Among Testicular Cancer Survivors
Source: Cancer Med. 2026 Feb 10;15(2):e71602. doi: 10.1002/cam4.71602 (PMC12890573; doi:10.1002/cam4.71602)
Supplement: Supplementary file 1 — Table S1: cam471602‐sup‐0001‐TableS1.docx. [file CAM4-15-e71602-s001.docx]

**Supplementary Table 1**

| Single episode Major depressive disorder mild, mod, severe w/out psychotic, severe w/ psychotic, unspecified | 296.21, 296.22, 296.23, 296.24, 296.2 | F32.0, F32.1, F32.2, F32.3, F32.9 | SSRI (1st line) - fluoxetine, sertraline, citalopram, escitalopram, paroxetine, and fluvoxamine  SNRI - venlafaxine, duloxetine, desvenlafaxine, levomilnacipran, and milnacipran  Serotonin modulator -  trazodone, vilazodone, and vortioxetine  Atypical - bupropion, mirtazapine  TCA - amitriptyline, imipramine, clomipramine, doxepin, nortriptyline, and desipramine  MAO inhibitor - tranylcypromine, phenelzine, selegiline, and isocarboxazid |
| --- | --- | --- | --- |
| Recurrent episode MDD mild, mod, severe w/out psychotic, severe w/ psychotic, unspecified | 296.31, 296.32, 296.33, 296.34, 296.3 | F33, F33.1, F33.2, F33.3, F33.9 | SSRI (1st line) - fluoxetine, sertraline, citalopram, escitalopram, paroxetine, and fluvoxamine  SNRI - venlafaxine, duloxetine, desvenlafaxine, levomilnacipran, and milnacipran  Serotonin modulator -  trazodone, vilazodone, and vortioxetine  Atypical - bupropion, mirtazapine  TCA - amitriptyline, imipramine, clomipramine, doxepin, nortriptyline, and desipramine  MAO inhibitor - tranylcypromine, phenelzine, selegiline, and isocarboxazi |
| Unspecified anxiety, generalized anxiety, mixed anxiety | 300, 300.02, 300.09 | F41.9, F41.1, F43.3 | SSRI -Citalopram Escitalopram Fluvoxamine Paroxetine Fluoxetine Sertraline  TCA - Doxepin Clomiprimine Nortriptyline Amitriptyline Maprotiline Desipramine Nortriptyline Doxepin Trimipramine Imipramine Protriptyline  MAO inhibitor - Isocarboxid Phenelzine Tranylcypromine  Azapirones - Buspirone  Benzodiazepines - Lorazepam Flurazepam Clonazepam Triazolam Chlordiazepoxide Temazepam Oxazepam Clorazepate Diazepam Alprazolam  Antihistamine - hydroxyzine  Noradrenergic blocker - Propranolol Atenolol Prazosin  ATypical - Aripiprazole Ziprasidone Risperidone Quetiepine Olanzapine |
| Suicidal Ideation | V62.84 | R45.851 |  |
